# Tetanus—Epidemic or Constitutional among Negroes?

**Published:** 1880-01

**Authors:** R. H. Goldsmith

**Affiliations:** Baltimore, Md.


ARTICLE V.

### TETANUS—EPIDEMIC OR CONSTITUTIONAL AMONG NEGROES?

BY R. H. GOLDSMITH, M. D., BALTIMORE, MD.

The following cases, occurring at various intervals, will serve to illustrate the marked predisposition of the African to tetanic spasms. The first case, a strong demonstration of the action of cold on the nervous system of the negro:

Antony, a plantation servant, ran into the woods, and was gone about two months. A few hours before I was summoned, he was found near the quarter perfectly rigid, insensible. I found him (12 o'clock at night) in strong spasm, the spine so completely arched that the patient's body touched the bed at only two points, the occiput and ossa calcës. There was also trismus, with utter inability to swallow. In the intervals between the spasms, he shrieked at the slightest touch; in fact, a sudden jarring of the bed would precipitate him into another attack. My treatment was as follows: Covered him with mustard plasters anteriorly, and applied a blister of cantharides from nape of neck to end of spine. Per rectum the following enema:

| | |
|---|---|
| ℞.—Quiniæ Sulph., | gr. xx. |
| Ol. Terebinth, | ℥ i. |
| Tr. Opii, | ʒ ij. |
| Aq. Amyli, | ℥ ji. |

Mix and give at once.

In three hours repeated same, with no apparent amelioration, and, in addition, ordered a foot-bath made strongly stimulant with cayenne pepper. Kept him in bath for fifteen minutes, and wrapped him in blankets. At six o'clock the blister was dressed, and I perceived a marked improvement. The spasms were not so frequent, the jaws relaxed, and he could swallow, although still with difficulty. Immediately made him swallow the following mixture:

| | | |
|---|---|---|
| ℞.—Morph. Sulph., | . . . . . . . . | gr. ss. |
| Spts. Vini Gallici, | . . . . . . | *f* ℥ i. |
| Quiniæ Sulph., | . . . . . . . | gr. x. |
| Aq., | . . . . . . . . . | ℥ i. |

Mix. To be repeated every two hours.

In the meantime, I had procured some chloroform, and as soon as there was a relaxation of spasm, gave ten drops with each of above doses. He was also made to inhale the chloroform, but never allowed to fall fully under its influence. This treatment was continued, with variations, for twenty-four hours, and, finally, with the happiest results. The spasms recurred more seldom, and the patient slept about four hours, when he was awakened, suddenly, by a stupid servant. Spasm recurred, but, to my delight, with much less severity and shorter duration. Continued morphine, quinine and chloroform, but now gave them in milk punch. The effect was most happy; the spasms returned at constantly lengthened intervals, until in three days after seeing the case the man was convalescent.

The second case, a boy, aged fourteen, while playing about a cotton gin, was caught by the right arm and dragged into the machinery. The limb was fractured in several places, and the soft parts torn from the bones. The elbow-joint was destroyed, the adjacent muscles either torn or bruised, and the brachial artery and nerve lacerated. I amputated at the middle third of the humerus, and the patient rallied kindly in four or five hours. Under the influence of opium he slept quietly. The case progressed happily until, the seventh day, a sudden change occurred in the atmospheric temperature, the thermometer fell about 20° in a few hours, and the boy in the act of drinking water was seized with tetanus, and his jaws suddenly clinched on the cup. I found him with the cup still between his teeth, suffering the greatest agony. The stump was fairly leaping in tetanic spasm. My treatment was, to cause him to inhale chloroform until the spasm relaxed, and then to deaden sensation of pain by keeping partially under the chloroform influence. As soon as he could swallow, gave the chloroform in ten-drop doses, internally, with morphine, until he slept. He recovered after five days' treatment.

In another instance, some plantation hands were rolling logs, and a negro man was crushed under the largest. I found him with a compound comminuted fracture of both tibia and fibula, with tibial bone protruding. Carefully reduced the fracture and brought the comminuted portions into as nice co-optation as the nature of the injury permitted. My apparatus was the wooden box in general use, filled with bran. The weather being very warm, I was compelled to cleanse the limb frequently. Five days after the accident tetanus occurred, and I was summoned. I could procure nothing but laudanum, turpentine and quinine, and gave these freely until I received some chloroform. For nearly eight days did this man linger between life and death. I finally extracted some spiculæ of bone, and he then commenced to improve. The spasms returned again and again during all that time, but were ever moderated by chloroform, until recovery was finally complete.

These three cases are typical of the extreme tendency, in the Southern States, to tetanus among the negroes. They illustrate the value of chloroform internally and by inhalation, and the result in each case shows the value of unremitting effort and perseverance in treatment. I had no hope of rescuing either party, as, according to statistics, nearly all tetanic cases die. But active effort changed statistics.

